# An assessment of Good Participatory Practice Guidelines at HIV prevention research clinical centers in Eastern and Southern Africa

**DOI:** 10.1186/1742-4690-9-S2-O25

**Published:** 2012-09-13

**Authors:** BP Ngongo, S Hannah, J Mbogua, M Seyoum, M Warren, E Baas, F Priddy

**Affiliations:** 1 International AIDS Vaccine Initiative, Nairobi, Kenya; 2 AVAC, New York, NY, USA

## Background

Compliance with ethical, regulatory, and scientific guidelines does not always guarantee consideration of external stakeholders’ concerns in research. In 2007, UNAIDS and AVAC: Global Advocacy for HIV Prevention developed the Good Participatory Practice (GPP) Guidelines to ensure community and stakeholder engagement in biomedical HIV prevention research. In 2010-2011, the International AIDS Vaccine Initiative (IAVI) and AVAC conducted the first comprehensive evaluation of perceptions, practices and recommendations for improvement of participatory practice at eight IAVI-sponsored research centers (RCs) in Eastern and Southern Africa.

## Methods

A total of 234 pre- and post-workshop questionnaires, 20 focus group discussions (FGD), and nine interviews with research staff, community advisory board members and other stakeholders were administered by AVAC staff primarily, to eliminate potential bias. STATA11 and ATLAS.ti were used to analyze the quantitative and qualitative data respectively.

## Results

Quantitative analysis showed a high level of baseline support regarding the relevance of 11 of 16 GPP focus areas. Lower relevance was placed at baseline on engagement in: protocol development, standards of HIV prevention, policies on non-HIV related harms, and trial accrual and follow-up. Support increased for all 16 GPP areas post-workshop with statistical significance levels varying between p<0.05 – p<0.01. Preliminary findings from qualitative data revealed that stakeholder engagement in formative research, advisory mechanisms, and informed consent processes were considered effective at most RCs. Areas of needed improvement included communication and transparency of stakeholder engagement activities, formalized documentation of GPP practices, and funding for GPP activities at RCs.

## Conclusion

Findings suggest that evaluation workshops significantly increased RC buy-in of GPP relevance to research. Some GPP areas may require more emphasis than others in future evaluations and technical assistance. On-site participatory GPP evaluations were highly effective in identifying strengths and gaps, increasing ownership, and facilitating ease of GPP implementation for research centers.

